# Interference of Quorum Sensing by *Delftia* sp. VM4 Depends on the Activity of a Novel *N*-Acylhomoserine Lactone-Acylase

**DOI:** 10.1371/journal.pone.0138034

**Published:** 2015-09-18

**Authors:** Vimal B. Maisuria, Anuradha S. Nerurkar

**Affiliations:** 1 Department of Microbiology and Biotechnology Centre, Faculty of Science, The Maharaja Sayajirao University of Baroda, Vadodara, Gujarat, India; 2 School of Life Sciences, Faculty of Health and Human Science, University of Hertfordshire, College Lane, Hatfield, Hertfordshire, United Kingdom; National Centre for Cell Science, INDIA

## Abstract

**Background:**

Turf soil bacterial isolate *Delftia* sp. VM4 can degrade exogenous *N*-acyl homoserine lactone (AHL), hence it effectively attenuates the virulence of bacterial soft rot pathogen *Pectobacterium carotovorum* subsp. *carotovorum* strain BR1 (Pcc BR1) as a consequence of quorum sensing inhibition.

**Methodology/Principal Findings:**

Isolated *Delftia* sp. VM4 can grow in minimal medium supplemented with AHL as a sole source of carbon and energy. It also possesses the ability to degrade various AHL molecules in a short time interval. *Delftia* sp. VM4 suppresses AHL accumulation and the production of virulence determinant enzymes by Pcc BR1 without interference of the growth during co-culture cultivation. The quorum quenching activity was lost after the treatment with trypsin and proteinase K. The protein with quorum quenching activity was purified by three step process. Matrix assisted laser desorption/ionization-time of flight (MALDI-TOF) and Mass spectrometry (MS/MS) analysis revealed that the AHL degrading enzyme (82 kDa) demonstrates homology with the NCBI database hypothetical protein (Daci_4366) of *D*. *acidovorans* SPH-1. The purified AHL acylase of *Delftia* sp. VM4 demonstrated optimum activity at 20–40°C and pH 6.2 as well as AHL acylase type mode of action. It possesses similarity with an α/β-hydrolase fold protein, which makes it unique among the known AHL acylases with domains of the *N*-terminal nucleophile (Ntn)-hydrolase superfamily. In addition, the kinetic and thermodynamic parameters for hydrolysis of the different AHL substrates by purified AHL-acylase were estimated. Here we present the studies that investigate the mode of action and kinetics of AHL-degradation by purified AHL acylase from *Delftia* sp. VM4.

**Significance:**

We characterized an AHL-inactivating enzyme from *Delftia* sp. VM4, identified as AHL acylase showing distinctive similarity with α/β-hydrolase fold protein, described its biochemical and thermodynamic properties for the first time and revealed its potential application as an anti-virulence agent against bacterial soft rot pathogen *Pectobacterium carotovorum* subsp. *carotovorum* based on quorum quenching mechanism.

## Introduction

Novel antimicrobials which specifically target the virulence factors are potentially revitalizing the pathogen management practices. Quorum sensing mechanism has been well studied in several gram-negative pathogens, such as *Pectobacterium* species, *Pseudomonas aeruginosa* and *Vibrio cholarae* [1,2]. Virulence gene expression in many Gram-negative pathogens generally is found to be under quorum sensing control [3–5]. In particular, quorum-sensing plays an important role in the regulation of virulence genes (mainly Plant Cell Wall Degrading Enzymes, PCWDEs production) in phytopathogenic soft rot causing *Pectobacterium* species. Also the *N*-acylhomoserine lactone (AHL) based quorum sensing in soft rot *Pectobacterium* species tightly regulates the activities implicated in microbial competition and host-pathogen interaction. These activities require various microbial functions such as biofilm formation, expression of virulence factors, antibiotic production etc. [6–11]. This mechanism of gene regulation could also presumably provide advantage to different competitors with quorum-sensing regulated functions in their natural surroundings.

Quorum sensing system is target of many antimicrobial strategies [12,13]. Enzymatic degradation of AHLs is generally the most common approach to quorum quenching [8,12,13,14]. Several soil bacteria producing AHL degrading enzymes have been characterized extensively for quorum quenching in past decade. Two major groups of AHL-degrading enzymes from bacterial species have been well characterized in literature [8,13]. One of which is AHL lactonase, a type of widely characterized AHL degrading enzymes, which was identified as *aiiA* in a *Bacillus* species and interferes with quorum sensing system of other bacteria by hydrolyzing the lactone ring of AHL [12,15–17]. Second type of AHL degrading enzyme found in many Gram-negative bacteria is AHL acylase which degrades the AHL signal molecules by hydrolyzing the amide linkage of AHL [18–20]. Bacterial system or in transgenic plants expressing AHL degrading enzymes show quorum quenching due to abolition of the quorum sensing regulated virulence and thereby infection [12,20]. The AHL acylase have advantage over AHL lactonase producing bacteria as the AHL degradation product of AHL acylase is homoserine lactone (HSL) and fatty acyl moiety that can be further metabolized as carbon, nitrogen and energy source by bacteria, while acyl homoserine, the product of AHL lactonase causes inhibitory effect on its growth to certain extent [15]. Altogether, studies have demonstrated that different AHL degrading enzymes of soil bacteria could efficiently counteract the quorum sensing regulation of bacterial pathogenicity and could be utilized as a new type of biocontrol mechanism [9,18,21,22].

It is well known that virulence of soft rot causing *Pectobacterium carotovorum* subsp. *carotovorum* (Pcc) is associated with the production of PCWDEs including pectate lyase, pectin lyase, polygalacturonase and cellulase [6,10,23]. Here in the present studies, an efficient AHL degrading soil isolate was evaluated for its interference on quorum sensing of soft rot causing Pcc strain BR1. The present study was undertaken with an aim of purification, identification and biochemical characterization of the AHL degrading factor.

## Materials and Methods

### Bacterial strains and culture conditions

All bacterial cultures were grown in their appropriate medium at 30°C under shaking condition with 140 rpm. Biosensor strain *Chromobacterium violaceum* CV026 was used to detect AHL [24,25]. Biosensor strain *C*. *violaceum* CV026 was grown overnight and maintained on 5.0 g L^-1^ Tryptone, 3.0 g L^-1^ Yeast extract (TY) medium supplemented with 20 μg mL^-1^ Kanamycin at 30°C. The *Escherichia coli* JM109 [F′*traD36 proAB lacIqlacZΔM15/recA1 endA1 gyrA96 thi hsdR17 supE44 relA1Δ (lac-proAB) mcrA*] bioluminescence biosensor strains based on *luxRI*’ [*pSB*401 for *N*-hexanoyl-homoserine lactone (HHL, Sigma-Aldrich), *N*-3-oxo-hexanoyl-L-HSL (OHHL) and *N*-3-oxo-octanoyl-L-HSL (OOHL, Sigma-Aldrich)] and *lasRI’* [*pSB*1075 for *N*-decanoyl-L-HSL (DHL, Sigma-Aldrich)] were used for AHL bioassay. These biosensor strains were grown and maintained in Nutrient Broth (NB per L: 5.0 g peptic digest of animal tissue, 5.0 g sodium chloride, 1.5 g beef extract, 1.5 g yeast extract and pH adjusted to 7.4 before sterilization) [26] amended with appropriate antibiotics (5 μg mL^-1^ Tetracycline for *E*. *coli* JM109 pSB401 and 40 μg mL^-1^ Ampicilin for *E*. *coli* JM109 pSB1075 at 30°C). As mentioned in previous studies the *P*. *carotovorum* subsp. *carotovorum* BR1 was grown and maintained on Nutrient broth supplemented with Pectin (NBP per L: 5.0 g peptic digest of animal tissue, 5.0 g sodium chloride, 1.5 g beef extract, 1.5 g yeast extract, and 5.0 g pectin, pH adjusted to 6.5 before sterilization) [27] at 30°C. AHL degrading bacterial isolates were initially grown on OHHL amended minimal medium and further maintained on NB.

### Isolation, characterization, identification and growth condition of AHL degrading bacteria

Turf soil samples were collected from the gardens of Faculty of Science, The M.S. University of Baroda, India. The medium reported by Leadbetter and Greenberg [19] was used for the enrichment and isolation of AHL-degrading bacteria. Growth substrates (2 mM HHL) were added to the autoclaved, vitamin-amended medium as indicated. At the end of enrichment cycle, colonies with diverse cultural characteristics were screened for the AHL-degrading properties in AHL-bioassay with biosensor strain *C*. *violaceum* CV026 [24,25]. The 16S rDNA based identification of specifically selected isolate for its high AHL degradation ability was performed as described previously [28]. Approximately 1,500 bp of the 16S rDNA was amplified using the universal eubacterial primers 27F (5′-GAGAGTTTGATCCTGGCTCAG) and 1492R (5′-TACCTTGTTACGACTT). The 16S rRNA gene sequence information of amplified fragment was obtained through the commercial sequencing services of Eurofins MWG GmbH, Ebersberg, Germany. The sequence data was matched using the tools provided at NCBI database (http://www.ncbi.nlm.nih.gov). The phylogenetic analysis was performed with this partial sequence using the default parameters provided at RDP database (http://rdp.cme.msu.edu). The branching pattern was analysed using 100 bootstrap replicates.

### Preparation of natural AHL extracts

Natural AHL was extracted according to Shaw et al. [29] from the culture supernatant of late exponential growth phase Pcc BR1culture. Cell-free supernatant was prepared by centrifugation at 14,000 ×g for 10 min at 4°C. AHL molecules were extracted thrice with equal volume of cold acidified ethyl acetate (0.01% v/v acetic acid) and concentrated using rotary evaporation. The concentrated AHL extract was dissolved in acidified ethyl acetate or acetonitrile and stored at -20°C. All synthetic AHL stock solutions were prepared at 1 mg mL^-1^ concentration in acidified ethyl acetate and stored at -20°C.

### AHL bioassays

For estimation of extracted AHL or that present in culture supernatant, violacein broth bioassay was used [30]. The purple color of *C*. *violaceum* CV026 pigment produced in response to the additions of spent medium extract or synthetic autoinducer was expressed as violacein units = (Abs_585nm_ / Abs_660nm_)×1000. To determine the concentration of AHL molecules, induction of bioluminescence in *E*. *coli* plasmid-borne *lux* sensors (pSB401 and pSB1075) was analyzed using Adavace Celsis Luminometer (Celsis, UK) and Glomax-Multi^+^Microplate Luminometer (Promega, UK). Microtitre plate assay for assessment of AHL activation of *E*. *coli* lux biosensor cells carrying reporter plasmids was performed as follows: Aliquots of the AHL being assayed were made in acetonitrile (HPLC grade, Sigma-Aldrich, UK) and dried overnight. 200μl of diluted *E*. *coli* lux biosensor cells were added to the dried AHL samples and the assay plates were incubated at 30°C, on a rocking platform for 4 h, and bioluminescence was measured using microplate Luminometer [31].

### Enzyme assays

Polygalacturonase (PG) and Pectate lyase (PL) activities were determined as described earlier [26,27]. AHL degrading activity was determined by measuring residual AHL concentration using bioluminescence based detection system. The purified protein solutions were mixed with 1:5 volumes of 125 μM AHL in 10 mM Phosphate Buffer Saline (PBS; pH 6.5) incubated at 30°C for 2 h. Residual AHL concentration was detected using *E*. *coli* lux (pSB401 or pSB1075) biosensors cells in Luminometer as mentioned in AHL bioassay as well as by HPLC. One unit of AHL degrading activity was defined as the amount of enzyme required to degrade 1 nM AHL molecules at 30°C and pH 6.5 per minute.

### Co-cultivation of Pcc BR1 and AHL degrading isolates

For co-cultivation experiments, Pcc BR1 and the selected AHL degrading isolates were grown overnight separately in NB at 30°C. PccBR1 was inoculated in NBP at 1.75 ×10^6^ CFU mL^-1^ along with AHL degrading isolates at 1.12–4.5 ×10^8^ CFU mL^-1^. All the co-culture combinations were performed in triplicate with incubation at 30°C under 140 rpm shaking condition. To analyze the growth rate, bacterial cells were enumerated as CFU by plating serially diluted samples in triplicate on Violet Red Bile Agar (VRBA) medium (HiMedia Laboratories Pvt. Ltd., India) and NB agar. The CFU counts of the two cultures could be separately recorded due to difference in colony morphology on the selective media [32]. Samples were withdrawn at 6 h for determination of AHL amounts in culture supernatant using AHL broth bioassay, while PG and PL activities were determined from culture supernatant as mentioned earlier.

### Purification and characterization of AHL degrading enzyme

Overnight grown cells of *Delftia* sp. VM4 were harvested from the culture broth by centrifugation at 12,000 rpm for 10 min at 4°C. Cell pellet was resuspended in 10 mM Tris-HCl (pH 6.5) containing 0.1 mM PMSF, and then homogenized using a cell sonicator. The precipitated proteins were collected from a 40–70% saturation of ammonium sulfate, dissolved in 10 mM Tris-HCl (pH 6.5), and dialyzed against the same buffer. Partial purification of the AHL-degrading enzymes was performed using anionic-exchange column chromatography (DEAE-sepharose CL-6B column, 0.8×12 cm) equilibrated with the 10 mM Tris-HCl (pH 6.5). The dialysate was applied to a column and eluted with NaCl gradient (0–1.0 M). The active fractions from DEAE-sepharose CL-6B column showing AHL-degrading enzyme activity were collected, concentrated by centrifugal concentrator tubes (Centriprep-10, Millipore), and loaded onto a Sephadex G50-80 column (1.5×50 cm) equilibrated with the 10 mM Tris-HCl (pH 6.5). Active fractions were pooled, concentrated and then mixed with sample buffer for native gel electrophoresis and resolved on 12% SDS-PAGE in duplicate as described by Park et al. [22]. Following electrophoresis, one of the gel was stained with silver salts [33] and the other was renatured by washing with 10 mM Tris-HCl (pH 6.5) containing 2.0% Triton X-100 for 15 min with gentle shaking at room temperature. The renatured gel was washed twice with 10 mM Tris-HCl (pH 6.5) then bands were sliced with reference to protein bands on the silver stained gel. The gel slices were mixed with 20 μM HHL, and incubated with gentle shaking at 30°C for 2 h. After the incubation, the reaction mixture was filtered through 0.45 μm nitrocellulose membrane filter and 30 μl filtrate was added to 170 μl of *C*. *violaceum* CV026 in TY broth medium (OD 600nm, 0.002) into a microtitre plate. Peptide mass fingerprinting analysis of the active protein band possessing AHL degrading activity was carried out at The Centre for Genomic Application (TCGA), New Delhi, India. The protein samples were processed for alkylation, reduction and tryptic digestion, the mass finger printing was done by MALDI-TOF MS and was also subjected to MS/MS analysis by LC-MS. Multiple amino acid sequence alignment of purified protein sample was carried out. Sequence alignment was performed with ClustalW (http://www.ddbj.nig.ac.jp/search/clustalw-j.html) and presented with Boxshade 3.21 (http://www.ch.embnet.org/software/BOXform.html). Prediction of putative secondary structure was done using a web-based service NetSurfP (http://www.cbs.dtu.dk/services/NetSurfP).

### Effect of temperature and pH on enzyme activity and kinetics

The temperature optima were determined by incubating an appropriate amount of enzyme with 125 μM HHL in 50 mM Phosphate buffer (pH 6.5) at various temperatures ranging from 20–70°C for 2h. The effect of pH on enzyme activity was determined by measuring activity at 30°C for 2 h, using different buffers: 50 mM Na-acetate buffer (pH 3.5, 4.15, 4.55, 5.0, 5.5, 6.0), 50 mM phosphate buffer (pH 6.0,6.2, 6.4, 6.6, 6.8, 7.0) and 50 mM Tris-HCl buffer (pH 6.6, 6.74, 7.0, 7.2, 7.4, 7.6). AHL bioassay was performed as mentioned above in section. The kinetic analysis of AHL degrading enzyme was performed according to Maisuria and Nerurkar [27].

### HPLC, EI-MS and NMR analyses of AHLs and its degradation product

AHL degradation products of purified AHL degrading enzyme reaction and AHLs were analyzed using reverse-phase HPLC, NMR and electron impact ionization-mass spectrometry (EI-MS) [11,22]. For the reverse-phase HPLC analysis, the sample was dissolved in 0.1 mL 50% (v/v) methanol (in MiliQ water) and analyzed using a C_18_ reverse-phase column (250×4.6mm S-4μm, 80A, YMC co. ltd. JH08S04-2546WT). The fractions were separated by isocratic gradient (1–100%) of water: formic acid (0.1%, pump A) and acetonitrile: formic acid (0.1%, pump B) at a flow rate of 0.3 mL min^-1^ detected by SPD-10UV detector at 210nm in HPLC unit (Shimadzu Corp., Japan). The EI-MS was performed using a DSQII GC/MS with TRACE GC Ultra unit (Thermo scientific), and high resolution ^1^H and ^13^C NMR was performed using Bruker NMR spectrometer. For MS analysis the sample was dissolved in 50% (v/v) methanol (in MiliQ water) and ionized by a positive-ion electron impact, while for NMR samples was dissolved in D_2_O. As a control standard, 5 mM of synthetic HHL and HSL (Sigma-Aldrich, India) dissolved in 50% (v/v) methanol (in MiliQ water) were used as AHL standards while the uninoculated medium was used as an additional control. The percentage of AHL degradation was determined by estimation of AHL with respect to known concentration that was loaded.

## Results and Discussion

### Isolated *Delftia* sp. VM4 can inactivate different types of AHLs

**Fig 1:**
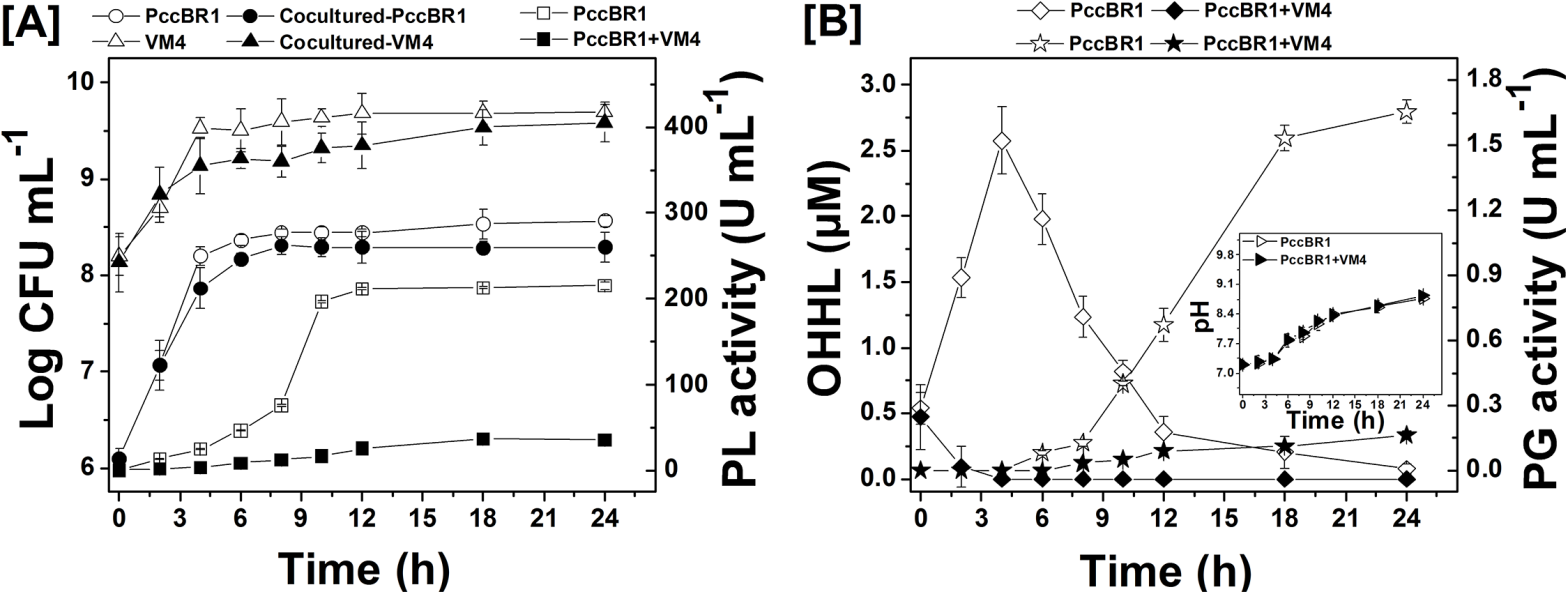
Effect of *Delftia* sp. VM4 on growth, AHL accumulation and virulence determinants production by PccBR1. [A] Time course of bacterial growth (circles, triangles) and PL production (squares). Pcc BR1 and *Delftia* sp. VM4 were incubated and grown separately (open symbols) or co-cultured (solid symbols). [B] AHL accumulation (diamonds), PG production (stars) and pH changes (triangles) in culture supernatant during co-culture of Pcc BR1 and *Delftia* sp. VM4. PccBR1 was inoculated alone (open symbols) or co-cultured (solid symbols). The experiment was repeated *n* = 3 times. The results are means ± SD (not shown where smaller than symbols).

Isolate VM4 showed consistently high AHL degrading activity in all AHL bioassay based screening and was identified as *Delftia* sp. based on its 16S rDNA partial sequence. Approximately 1000 nucleotide sequence of 16S rRNA gene (GenBank accession number JF717756) showed 100% identity to *Delftia tsuruhatensis* on BLAST search at NCBI database. This isolate VM4, on phylogentic analysis using 16S rRNA gene sequences, clustered along with *Delftia tsuruhatensis*, *Delftia lacustris* and *Delftia acidovorans* strains (S1 Fig), therefore, henceforth designated as *Delftia* sp. VM4. An AHL-degrading soil isolate belonging to genus *Delftia* previously reported by Jafra et al. [21] was found to inactivate exogenous AHLs supplemented in growth medium and attenuate the maceration of potato tissue caused by *P*. *carotovorum*. This inactivating mechanism was attributed to an unknown factor involved in AHL degradation. The purification and detailed investigation of AHL degradation activity from *Delftia* sp. has not been reported hitherto, which we aimed to do in this study. The effect of *Delftia* sp. VM4 on the growth, AHL accumulation and virulence determinant enzyme production of Pcc BR1 when analyzed in the co-culture condition showed that the PL production of Pcc BR1 was significantly inhibited by *Delftia* sp. VM4 without affecting its growth (Fig 1A). It reduced the OHHL accumulation significantly which resulted in suppression of virulence determinant enzymes (PG and PL) production of Pcc BR1 during co-culture (Fig 1A and 1B). The pH of the medium monitored intermittently was seen to increase slightly above neutrality. At 6 h of growth, the medium pH increased from 7.2 to 7.8, might not be responsible for decline in OHHL production as its accumulation was inhibited within 6 h during co-culture (Fig 1B). *Delftia* sp. VM4 interferes with quorum-sensing signaling of Pcc BR1 when they live commensally *in vitro*, and such signal interruption causes drastic attenuation of Pcc BR1 virulence determinants. Furthermore, *Delftia* sp. VM4 did not produce an antibiotic-like material to hinder the growth of Pcc BR1. This result supports the possibility of involvement of quorum quenching factor in AHL degradation by this selected isolate *Delftia* sp. VM4 preventing virulence of Pcc BR1.

The residual amount of HHL or OOHL in the culture medium significantly decreased with increase in cell density when the growth of *Delftia* sp. VM4 was measured with HHL and OOHL individually as the sole source of carbon and energy. Most of the HHL and OOHL in the culture medium were consumed after 10 h of cultivation (S2A Fig). No growth was observed in minimal medium without AHL. It has been reported that the half-life of AHL is less than 3 h under alkaline conditions (pH >8.0) [19]. During 10 h of cultivation in monoculture of *Delftia* sp. VM4, the pH of the culture medium increased to 6.7 (S2A Fig). Thus, the dramatic decrease of AHLs in the medium was unlikely to have been due to action of pH. Clearly, *Delftia* sp. VM4 was able to utilize HHL and OOHL as both the sole carbon and nitrogen source. This strain effectively degraded different types of AHL molecules (HHL, OHHL, OOHL and DHL) based on biosensor assay (S2B Fig). In particular, OHHL was degraded efficiently within 60 min, while DHL degradation was poor as even after 120 min the degradation was incomplete. The quorum quenching activity of *Delftia* sp. VM4 was lost after treatment with trypsin and proteinase K (S2B Fig). These observations suggest that the AHL degradation of *Delftia* sp. VM4 could be enzymatic reaction. Similar characteristics of AHL degrading activity of other Gram-negative bacterial strains such as *V*. *paradoxus* VAI-C and *P*. *aeruginosa* PAO1 in minimal medium supplemented with AHLs as sole energy source have been reported by other groups [19,34].

### Purification and characterization of AHL degrading enzyme of *Delftia* sp. VM4

**Fig 2:**
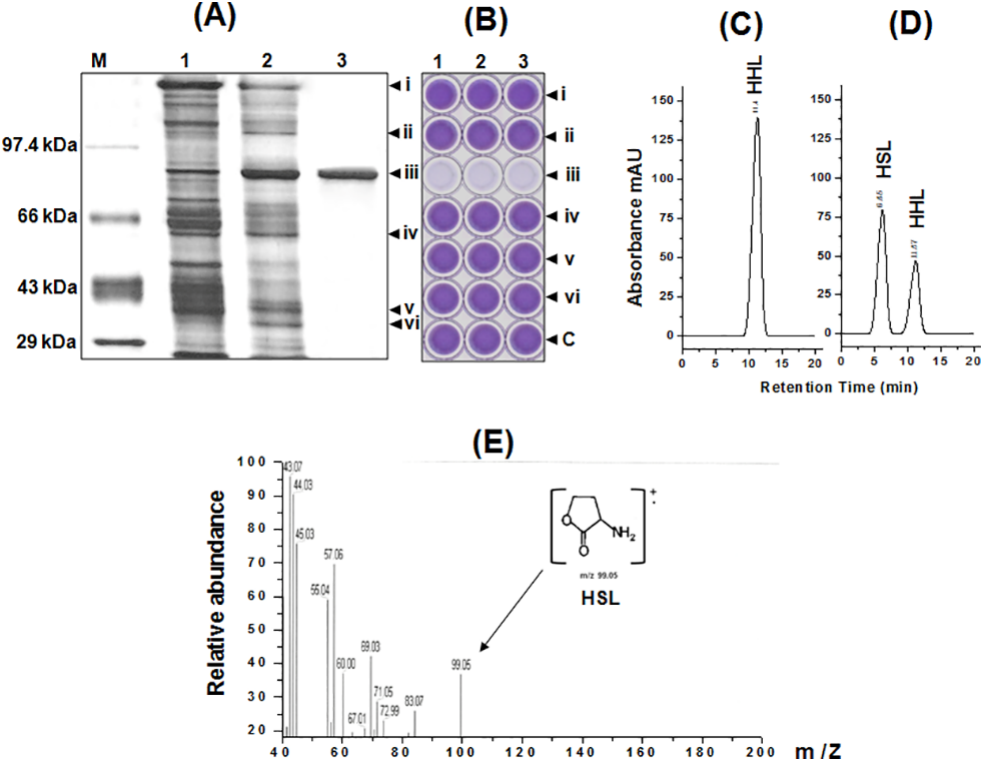
Characterization of AHL-acylase of *Delftia* sp. VM4. (A) Silver stained 12% SDS-PAGE during purification of AHL acylase from *Delftia* sp. VM4. Lane: M, PMWH- protein marker; 1, ammonium sulphate precipitates; 2, eluate from DEAE-sepharose column; 3, eluate from Sephadex column. (B) Microtitre plate assay for the AHL degrading activity with 0.05 mM HHL of the renatured gel slices (bands i-vi) from the SDS-PAGE was assayed using *C*. *violaceum* CV026 biosensor based bioassay. HHL (0.05 mM) was loaded as the control (well-c). HPLC profile of (C) HHL without purified AHL acylase, (D) HHL degradation by purified AHL acylase, (E) EI-MS analysis of degraded product (HSL) from HHL.

The AHL degrading activity of *Delftia* sp.VM4 was observed only in cell lysates, indicating that the enzyme is an intracellular protein. Extracellular activity in the supernatant was absent, thus the AHL-degrading enzyme was purified from the cell lysate using DEAE- sepharose CL6B and Sephadex G50-80 column chromatography. The final step of purification on Sephadex G50-80 column chromatography gave 155.8 fold purification and 12% yield of the enzyme (S1 Table). The active fractions with HHL degrading activity eluted from column chromatography were concentrated and separated on 12% SDS-PAGE. The purified fraction from gel permeation step gave a single band of 82 kDa size (Fig 2A). The enzyme activity assay of individual intense protein bands separated on SDS-PAGE was carried out with renatured gel slices (Fig 2B). The single protein band of about 82 kDa size (marked as band-iii, Fig 2A) exhibited AHL degrading activity as shown in corresponding wells of microtitre plate (Fig 2B). Bands labeled as i, ii, iv, v and vi of elute from DEAE-sepharose column were also tested in microtitre well plate assay shown in corresponding wells. Only protein band-iii of 82 kDa size possessed AHL degrading activity and was identified as AHL acylase type since it produced the product characteristic of HHL cleavage which was identified as HSL in HPLC (Fig 2C and 2D) and EI-MS (Fig 2E). The EI-MS analysis of the product revealed abundance of HSL with M+H ion at m/z 99.05 signifying that the cleavage of HHL (total M+H ion m/z 199 and identity M+H ion m/z 143) by the action of AHL degrading enzyme produced two products viz. HSL and acyl-moiety. Moreover, HSL structural information of four carbon ring structure was validated using ^13^C and ^1^H NMR as shown in S3A and S3B Fig. In order to identify the protein, fragments of tryptic digest of the protein eluted from the band (82 kDa on SDS-PAGE) were subjected to MALDI-TOF and MS/MS analyses. The spectrum obtained was matched with proteins in the NCBI GenBank database, using the Mascot search program. The search yielded a top score of 83.6 for hypothetical protein Daci_4366 [protein scores greater than 67 are significant at P<0.05 according to Xu et al. [35]]. Furthermore, the selected tryptic peptides of 901.39 m/z, 1008.46 m/z and 1630.84 m/z sequenced by MS revealed an amino acid sequence of GEWNLQR, DIQPMLHR and TTQLQNTEPLLAFR respectively, corresponding to residues of hypothetical protein Daci_4366 (gi| 5749954) of *Delftia acidovorans* SPH-1 (S4 Fig). The selected tryptic peptide of 1630.84 m/z further sequenced by MS/MS revealed the identical amino acid sequence as above, and the Mascot search yielded a top protein score of 177 for the hypothetical protein Daci_4366.

**Fig 3:**
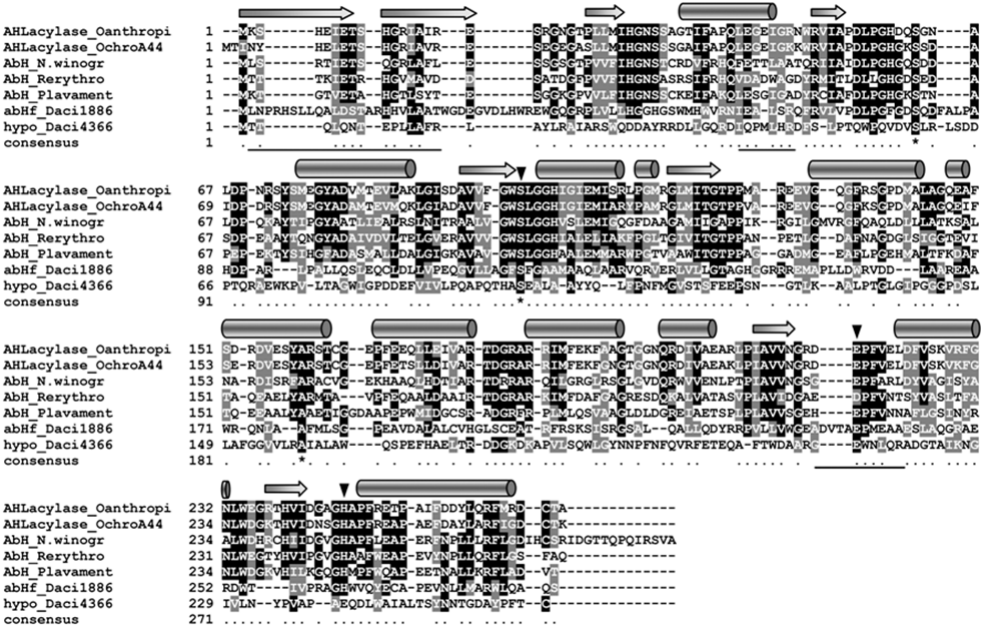
Multiple amino acid sequence alignment for AHL-acylase of *Delftia* sp. VM4 with AHL-acylases and α/β-hydrolases. In addition to hypothetical protein (Daci_4366, indicated as hypo_Daci4366, gi| 5749954) from *Delftia acidovorans*, acylase homologues used in the alignment includes, *Ochrobactrum* anthropi ATCC 49188 (AHLacylase_Oanthropi, gi|151560055), *Ochrobactrum* sp. A44 (AHL acylase_OchroA44, gi|298370747), and α/β hydrolase fold homologues includes, *Nitrobacter winogradskyi* Nb-255 (AbH-N.winogr, gi| 74419560), *Rhodococcus erythropolis* SK121 (AbH-Rerythro, gi|229491600), *Parvibaculum lavamentivorans* DS-1 (AbH_Plavament, gi|154156608) and *D*. *acidovorans* SPH-1 (Daci_1886, gi| 5747443, indicated as abHf_Daci1886). White letters on a black background depict identical residues, and similar residues are depicted by black letters on a gray background and gaps are represented by dashes to facilitate alignment. Conserved residues of relevance to autoproteolysis and catalysis in known acylases are marked with asterisks. ‘*’, identical residue; ‘dot’, semi-conserved substitution; ‘arrow’, putative β strands subunits; ‘tube shape’, putative α helices subunits. Residues positions known or thought to be possessed conservative amino acid residues (S, H, D/E) typical for the catalytic site of an α/β hydrolase are marked by triangles.

**Fig 4:**
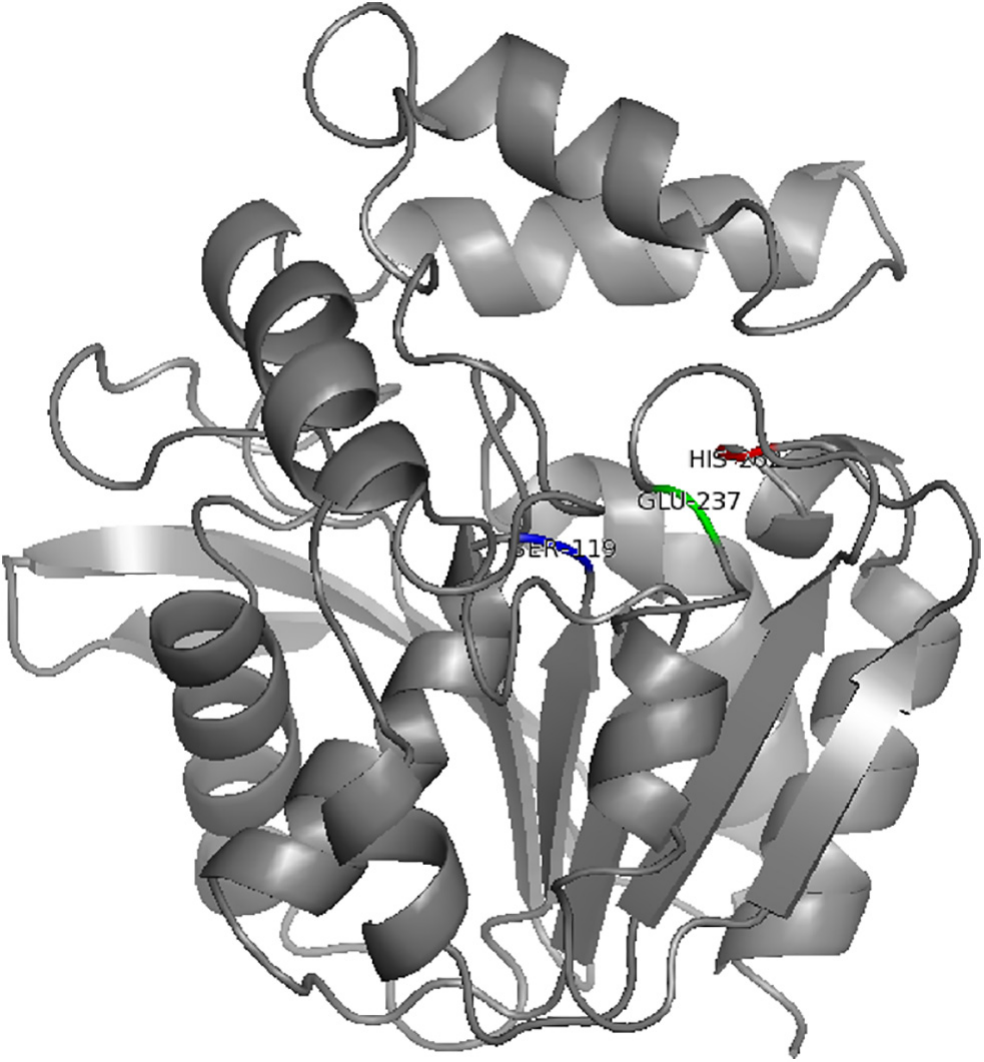
Predicted structural model of α/β-hydrolase fold protein of *Delftia*. Residue positions of known or deduced conserved amino acid residues (S, H, D/E) typical for the catalytic site of an α/β hydrolase are marked in blue, red and green respectively.

Interestingly, up till now the AHL acylases from *Streptomyces* sp. M664 [36], *Ralstonia* sp. XJ12B [20], *Shewanella* sp. MIB015 [37] and *Anabaena* sp. PCC7120 [38], PvdQ [34] and QuiP [39] from *P*. *aeruginosa*, HacA and HacB from *P*. *syringae* [40] were reported to contain domains characteristic of the Ntn-hydrolase (Ntn: N-terminal nucleophile) superfamily which are described as those possessing a wide variety of hydrolytic activities and distinct folds. In contrast, the purified enzyme (82 kDa) of *Delftia* sp. VM4 under study here possessed similarity with an α/β-hydrolase fold protein. This type of AHL acylase owning α/β-hydrolase fold was already identified in *Ochrobactrum* sp. A44 [41], which belonged to the carboxylic ester hydrolases (EC 3.1.1) as it possesses a nucleophile-His-Acid catalytic triad characteristic of typical carboxylic ester hydrolase [42]. An α/β-hydrolase fold protein was first time described by Ollis et al. [43]. The 3D-Jury structure based analysis using BioinfoMetaServer revealed that the hypothetical protein Daci_4366 has structural similarity with different Ntn-hydrolases. Inspection of genome database of *Delftia acidovorans* SPH-1, revealed α/β-hydrolase fold homologue (Daci_1886) that was also similar to reported α/β-hydrolase type AHL acylase of *Ochrobactrum* sp. A44 [41] and other homologues. The similarity of hypo_Daci4366 to the carboxylic ester hydrolases implies the presence of a catalytic triad composed of His, Asp or Glu and Ser (Fig 3). In summing up, it can be said that the purified enzyme from *Delftia* sp. VM4 represents a novel class of AHL-inactivating acylases. The constructed structure of α/β-hydrolase fold protein of *Delftia* predicted using online tool Easypred and processed with PymoL software is shown in Fig 4.

### Biochemical properties of purified AHL acylase from *Delftia* sp. VM4

**Fig 5:**
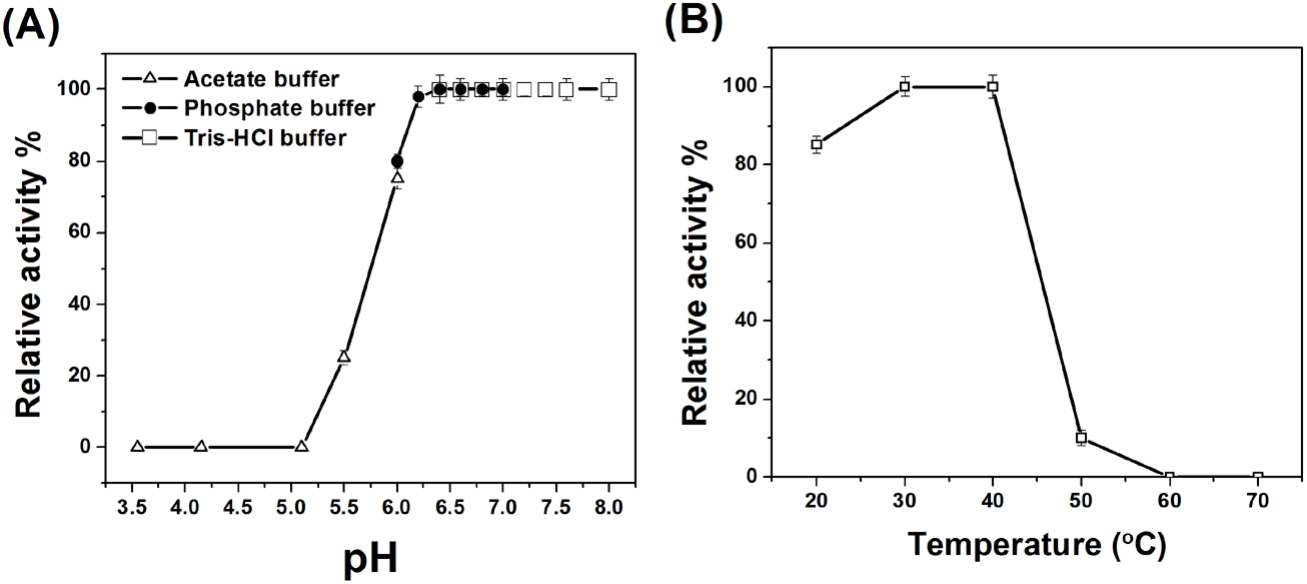
Effect of (A) pH and (B) temperature on purified AHL-acylase actvity of *Delftia* sp. VM4.

Purified AHL acylase activity was examined with HHL as substrate. The optimal pH for the AHL-degrading activity of purified AHL acylase was examined using HHL as substrate at 30°C. The AHL acylase activity was enhanced with increasing pH (above pH 5) and reached maximum at pH 6.2 (Fig 5A). At above pH 7.5, HHL is possibly inactivated by alkylation [44]. There is sparse information available in literature for optimum temperature and pH of AHL acylase. In contrast to this, AHL lactonase (AiiM) from *Microbacterium testaceum* StLB037 is reported as showing maximum activity at 37°C and pH 8.0 [45]. The activity of purified AHL acylase was (optimum more than 80%) at temperature range of 20–40°C, which was drastically decreased at elevated temperatures 50 and 60°C (Fig 5B). The kinetic and thermodynamic parameters for hydrolysis of AHL substrates (OHHL, OOHL and HHL) by purified AHL acylase from *Delftia* sp. VM4 were determined (Table 1) as described in our previous study [27]. This AHL acylase showed higher affinity with OHHL and OOHL compared to HHL as adjudged from lower *K*_m_ values. Also thermodynamic parameters for substrate hydrolysis of purified AHL acylase supported this observation (Table 1). There is no information available regarding kinetic and thermodynamic parameters of any AHL acylase in literature. However, these kinetic properties have been described by Wang et al. [46] for AHL-lactonase (AiiA) of *Bacillus* sp. In comparison to this AHL-lactonase (AiiA), the purified AHL acylase of *Delftia* sp. VM4 was found to be catalytically efficient for HHL, OHHL and OOHL hydrolyses. The *K*_m_, *k*_cat_ and *k*_cat_/*K*_m_ values of AHL-lactonase (AiiA) were reported as 3.83 mM, 35.67 sec^-1^, 9.31 mM^-1^ sec^-1^ respectively for HHL hydrolysis; and 2.28 mM, 22.17 sec^-1^, 9.72 mM^-1^ sec^-1^ respectively for OOHL hydrolysis. If the *k*_cat_/*K*_m_ values for the hydrolysis of HHL, OHHL and OOHL substrates by the purified AHL acylase of *Delftia* sp. VM4 (Table 1) is compared with *k*_cat_/*K*_m_ values of other wild-type lactonase homologues (*Vmo*Lac, GKL and MCP) [47–49], the purified AHL acylase ranks the highest. This can be attributed to elevated catalytic efficiency (*k*_cat_/*K*_m_) with effective substrate affinity (lower *K*_m_ values) and turnover number (higher *k*_cat_ values) (Table 1). The cations including Na^+^, K^+^, Mg^2+^ and Ca^2+^, showed no effect on AHL acylase activity at 2 mM concentration (Table 2). On the other hand, purified AHL acylase was partially inhibited by Ba^2+^, Mn^2+^, Ni^2+^ and Zn^2+^ at 1 mM and completely inhibited by Cd^2+^, Cu^2+^ and Pb^2+^ (Table 2).

**Table 1:** Michaelis-Menten constants and thermodynamic parameters for substrate hydrolysis of purified AHL acylase from *Delftia* sp. VM4.

AHLs	Kinetic Parameters^†^		Thermodynamic parameters	
HHL	*K*_m_ ^a^	1.25 mM	ΔH*	2.46 kJ/mol
	V_max_	404 U mg^-1^	ΔS*	-146.83 J/mol/ K
	*k*_cat_ ^b^	33.2 sec^-1^	ΔG* (free energy for activation)	48.42 kJ/mol
	*k*_cat_/*K*_m_ ^c^	26.6 mM^-1^ sec^-1^	ΔG* _E-S_ (free energy for substrate binding)	0.58 kJ/mol
	V_max_/*K*_m_ ^d^	0.727	ΔG*_E-T_ (free energy for transition state formation)	2.12 kJ/mol
OOHL	*K*_m_ ^a^	0.67 mM	ΔH*	2.46 kJ/mol
	V_max_	296.6 U mg^-1^	ΔS*	-149.4 J/mol/ K
	*k*_cat_ ^b^	24.3 sec^-1^	ΔG* (free energy for activation)	49.23 kJ/mol
	k_cat_/*K*_m_ ^c^	36.3 mM^-1^ sec^-1^	ΔG* _E-S_ (free energy for substrate binding)	-1.06 kJ/mol
	V_max_/*K*_m_ ^d^	1.0	ΔG*_E-T_ (free energy for transition state formation)	1.3 kJ/mol
OHHL	*K*_m_ ^a^	0.5 mM	ΔH*	2.46 kJ/mol
	V_max_	278 U mg^-1^	ΔS*	-149.9 J/mol/ K
	*k*_cat_ ^b^	22.8 sec^-1^	ΔG* (free energy for activation)	49.39 kJ/mol
	*k*_cat_/*K*_m_ ^c^	45.6 mM^-1^ sec^-1^	ΔG* _E-S_ (free energy for substrate binding)	-1.8 kJ/mol
	V_max_/*K*_m_ ^d^	1.25	ΔG*_E-T_ (free energy for transition state formation)	0.71 kJ/mol

^† a^Michaelis constant for substrate affinity

^b^Turnover number

^c^Second-order rate constant

^d^Catalytic efficiency

**Table 2:** Effect of cations on purified AHL acylase activity of *Delftia* sp. VM4.

Cations	AHL acylase activity (%)
AHLacylase control (No cations)	100
2mM Na^+^	100
2mM K^+^	100
2mM Mg^2+^	100
2mM Ca^2+^	100
1mM Ba^2+^	74
1mM Mn^2+^	63
1mM Ni^2+^	52
1mM Zn^2+^	27
1mM Cd^2+^	0
1mM Cu^2+^	0
1mM Pb^2+^	0

In summary, the present study deals with an efficient AHL degrading soil bacterium *Delftia* sp. VM4 capable of hydrolyzing different types of AHLs and by virtue of this enzyme it could afford protection against the virulence of Pcc BR1. The present study supported that the efficacy of *Delftia* sp. VM4 in preventing the production of virulence determinants in Pcc BR1 depends upon its ability to produce AHL acylase that inactivates AHL molecules by hydrolyzing amide bond between lactone ring and fatty acyl moiety. To the best of our knowledge, this AHL acylase belonged to a novel α/β hydrolase family fold, while all previously known AHL acylases belonged to amidase family. Further research on such AHL acylase would help in developing a new class of potential anti-virulence approach with broad spectrum activity. The AHL acylase from *Delftia* sp. VM4 showed efficient biochemical properties for the degradation of AHL signal and thus can be a useful anti-virulence agent against many bacterial pathogens where virulence is regulated by AHL type quorum sensing. Moreover, analysis on the application of this novel AHL acylase against many pathogens *in vivo* or *in situ* should not be ignored.

## Supporting Information

S1 Fig Phylogenetic analysis of isolate VM4 within the genus *Delftia*. The branching pattern was generated using Weighbor joining method. The Genbank accession number of the 16S rDNA sequences are indicated in parenthesis. The number at each branch indicates the bootstrap values out of 100. *Delftia litopenaei* is used as out-group.
(TIF)

S2 Fig [A] Time course of growth of *Delftia* sp. VM4 in minimal medium containing HHL or OOHL as a sole carbon source. Culture growth in the medium with HHL (filled circle), OOHL (filled triangle) and without AHL (open square) was assessed based on the O.D. at 600nm. The residual HHL (open circle) and OOHL (open triangle) concentration in the culture was measured using a bioassay. The pH change in culture supernatant was monitored during growth curve. [B] Inactivation of various AHLs by a cell suspension of *Delftia* sp. VM4. Equal volumes of a diluted cell suspension (OD600nm 1.0) and a PBS (pH-6.5) containing 100 μM of each synthetic AHLs (HHL, OOHL, DHL) and OHHL (extracted AHLs produced by Pcc BR1) were mixed and incubated at 30°C. For the experimental control a culture suspension was first treated with trypsin and proteinase K, and then mixed with 100 μM OOHL. Experiments were repeated n = 3 times; the results shown are means ± S.D.
(TIF)

S3 Fig (A) Proton-decoupled ^13^C-NMR spectrum of HHL degraded product (i.e. HSL) by purified AHL acylase of *Delftia* sp. VM4. The spectrum was measured in D_2_O at 100 MHz (chemical shifts in p.p.m. from tetramethylsilane) and the following data were obtained: 174.42 C-1, 67.24 C-4, 48.43 C-2, and 26.63 C-3. (B) High-resolution ^1^H-NMR spectrum HHL degraded products (i.e. HSL) by purified AHL acylase of *Delftia* sp. VM4. The spectrum was measured in D_2_O at 400 MHz (chemical shifts in p.p.m. from tetramethylsilane) and the data compared with standard HSL spectrum, indicate identical values.
(TIF)

S4 Fig Tryptic digest fingerprint and sequence matches after MALDI-TOF MS/MS analysis of purified AHL acylase from *Delftia* sp. VM4. (TIF)

S1 Table Purification of AHL acylase from *Delftia* sp. VM4. (PDF)
