# Supplementary figures and images for: Interference of Quorum Sensing by Delftia sp. VM4 Depends on the Activity of a Novel N-Acylhomoserine Lactone-Acylase
Source: PLoS One. 2015 Sep 18;10(9):e0138034. doi: 10.1371/journal.pone.0138034 (PMC4575145; doi:10.1371/journal.pone.0138034)

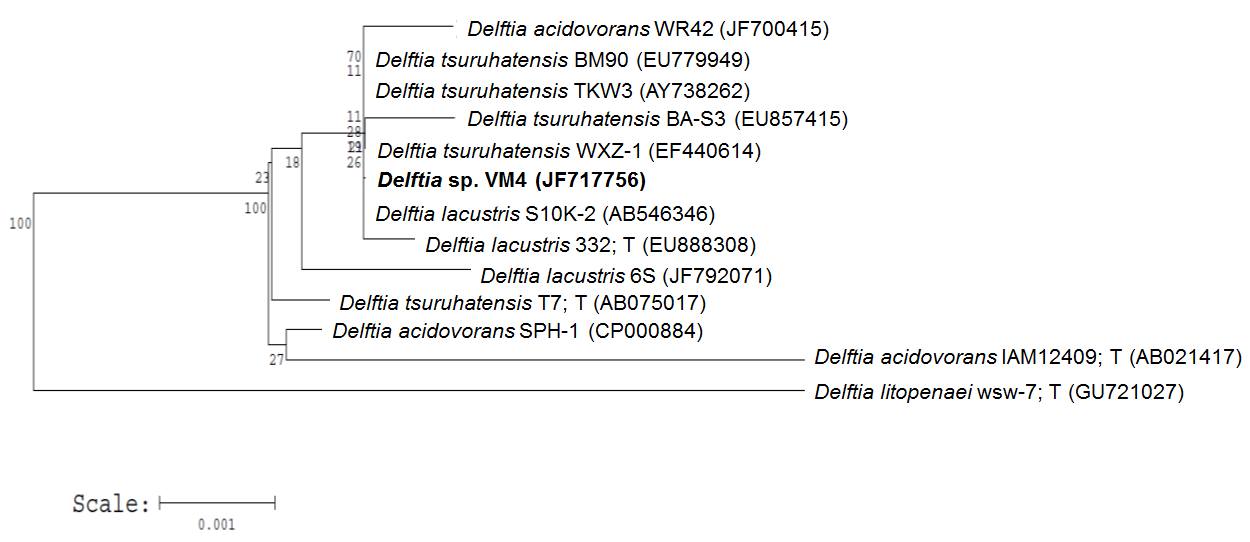

Supplement: S1 Fig — The branching pattern was generated using Weighbor joining method. The Genbank accession number of the 16S rDNA sequences are indicated in parenthesis. The number at each branch indicates the bootstrap values out of 100. Delftia litopenaei is used as out-group. (TIF) [file pone.0138034.s001.tif]

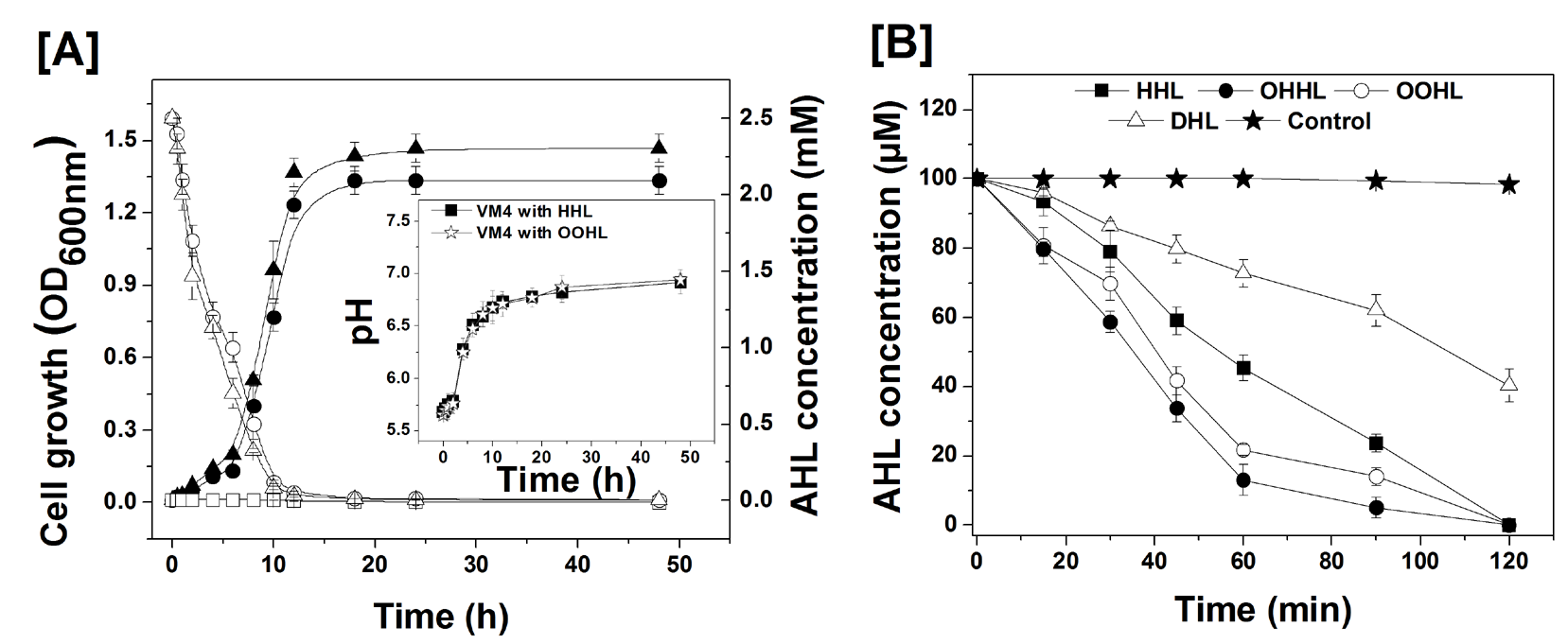

Supplement: S2 Fig — Equal volumes of a diluted cell suspension (OD600nm 1.0) and a PBS (pH-6.5) containing 100 μM of each synthetic AHLs (HHL, OOHL, DHL) and OHHL (extracted AHLs produced by Pcc BR1) were mixed and incubated at 30°C. For the experimental control a culture suspension was first treated with trypsin and proteinase K, and then mixed with 100 μM OOHL. Experiments were repeated n = 3 times; the results shown are means ± S.D. (TIF) [file pone.0138034.s002.tif]

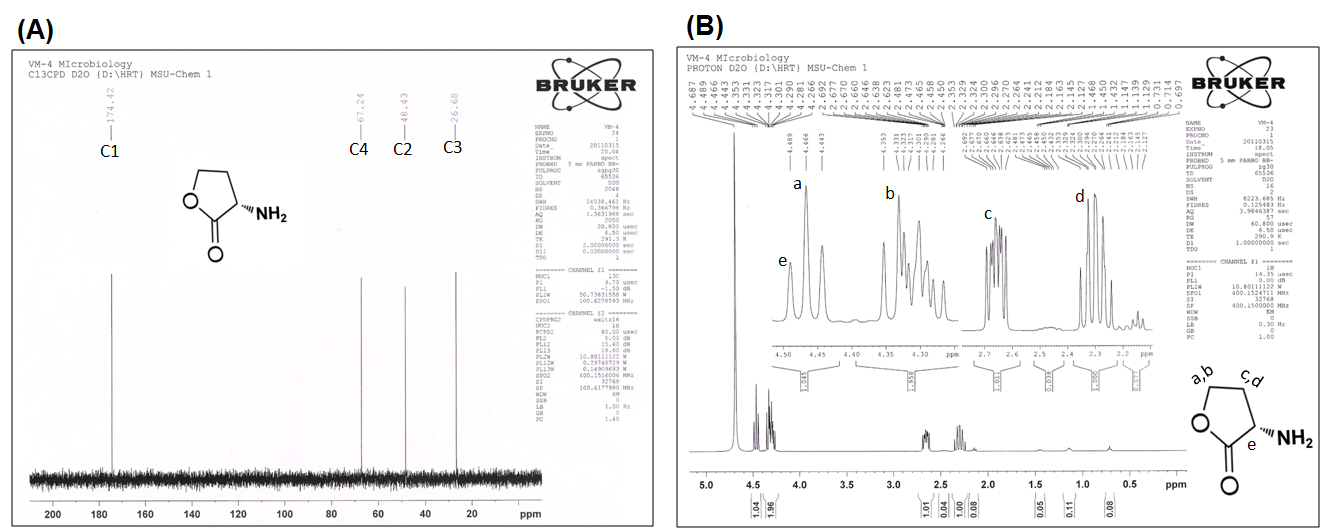

Supplement: S3 Fig — The spectrum was measured in D2O at 400 MHz (chemical shifts in p.p.m. from tetramethylsilane) and the data compared with standard HSL spectrum, indicate identical values. (TIF) [file pone.0138034.s003.tif]

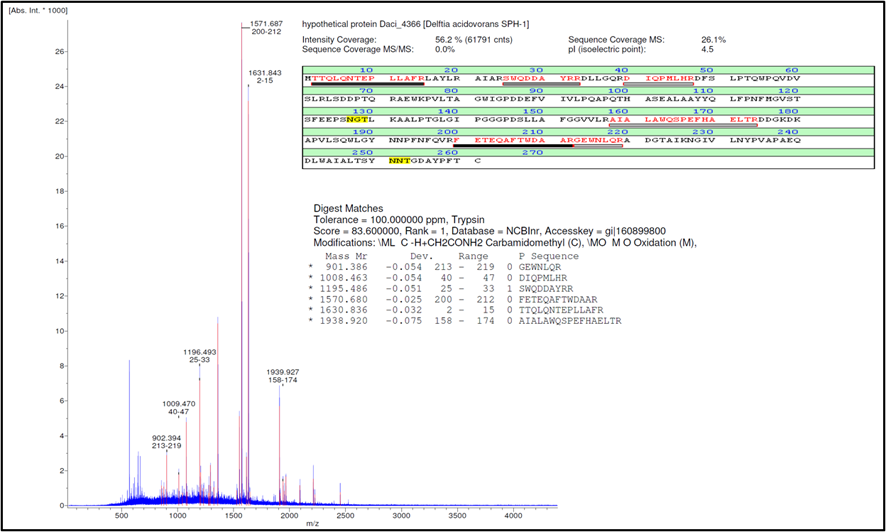

Supplement: S4 Fig — (TIF) [file pone.0138034.s004.tif]
