# Supplementary material for: Interference of Quorum Sensing by Delftia sp. VM4 Depends on the Activity of a Novel N-Acylhomoserine Lactone-Acylase
Source: PLoS One. 2015 Sep 18;10(9):e0138034. doi: 10.1371/journal.pone.0138034 (PMC4575145; doi:10.1371/journal.pone.0138034)
Supplement: S1 Table — (PDF) [file pone.0138034.s005.pdf]

Table S1. Purification of AHL acylase from *Delftia* sp. VM4.

Purification step	Total Protein (mg)	Total activity (Units)	Specific activity U mg^{-1}	Fold purification	Yield %
Crude	811.5	1000	1.23	1	100
Ammonium sulphate	259	505	1.95	1.6	51
DEAE-Sepharose CL6B	16.7	336	20.1	16.4	34
Sephadex G-50-80	0.63	120	191.6	155.8	12
